# False-positive FDG PET CT Scan in Vertebral Hemangioma

**DOI:** 10.22038/AOJNMB.2018.12010

**Published:** 2019

**Authors:** Shrikant Vasantrao Solav, Shailendra Vasant Savale, Abhijit Mahaveer Patil

**Affiliations:** SPECT Lab, Nuclear Medicine Services, Pune, Maharashtra, India

**Keywords:** Bone scan, CT, FDG PET CT, Tumor induced osteomalacia, Vertebral hemangioma

## Abstract

FDG PET CT scan is considered to be a sensitive tool to detect skeletal metastasis in known malignancies. However, it’s high sensitivity and low specificity may account for false positive diagnosis in cases of trauma, infection, inflammation and other benign conditions. Skeletal hemangioma is one of the common benign conditions which are typically ametabolic on FDG PET CT with no uptake on bone scan. However, rarely they may have atypical imaging features and appear hypermetabolic. Other imaging modalities such as MRI and CT scan have typical imaging findings for hemangioma and can be used for evaluation of focal hypermetabolic skeletal lesions. There are atypical imaging characteristics in each of these modalities. Hence when used judiciously they can complement each other and avoid a false positive test result. This case report highlights the importance of bone scan and CT scan in excluding pathological involvement of skeleton with false positive FDG PET scan result.

## Introduction

^18^F-fluorodeoxyglucose positron emission tomography- computed tomography (^18^F-FDG PET CT) is an established method for staging of carcinomas. Bone scan is an old method to detect skeletal metastasis. Both the modalities are usually normal in vertebral hemangiomas. However, there have been case reports of increased osteoblastic activity on bone scan with normal metabolic activity on PET scan in vertebral hemangiomas and vice versa.

Thus, in a suspected vertebral hemangioma it is important to recognize that either of these tests may be falsely positive. Discordance between FDG and bone scan in presence of radiologic (CT or MRI) features of hemangioma, may prevent an unnecessary invasive procedure.

## Case report

A 51 years old man with poorly differentiated tonsillar carcinoma had well defined enhancing hypodense mass in left tonsillar fossa measuring 36×29×58 mms with neck nodes on CT scan. Biopsy from neck nodes showed metastasis from squamous cell carcinoma. Patient was treated with radiotherapy using Intensity-Modulated Radiation Therapy (IMRT) technique to administer 7000 cGy in 35 fractions. Concurrent weekly Cisplatin was administered intravenously in the dose of 40 mg/square meter body surface area.

**Figure 1:**
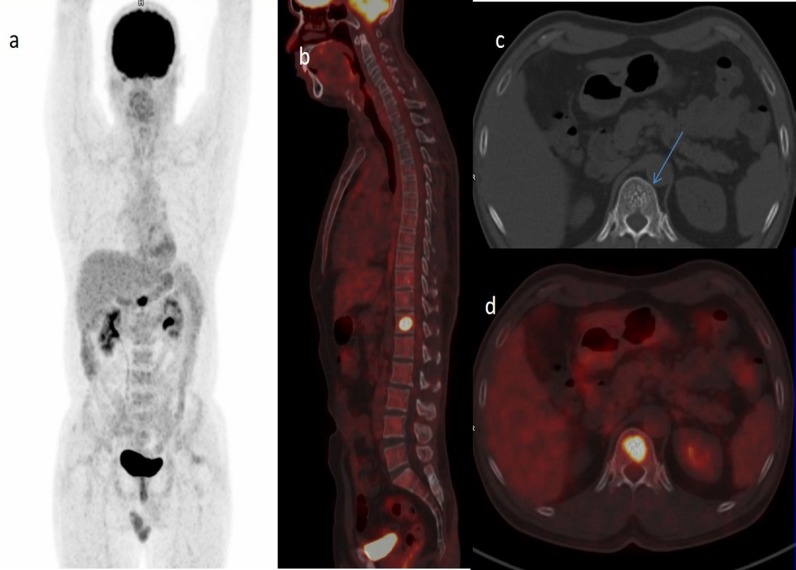
a, b and d) showing FDG avid lesion in T12 vertebra, c) showing classical polka dotted appearance of plain CT images

Post treatment PET/CT was performed at 1 hour after intravenous administration of 6.8 mCi ^18^F-FDG on 6 hours fasting state. Images were acquired using 16 slice time of flight biograph horizon scanner from Siemens. Left tonsillar fossa-base of tongue-lateral oropharyngeal wall were free of FDG avid lesions or cervical nodes suggesting response to treatment. Axial CT images showed ‘polka dot’ appearance in 12^th^ thoracic vertebra suggestive of hemangioma (Figure1c). 

**Figure 2:**
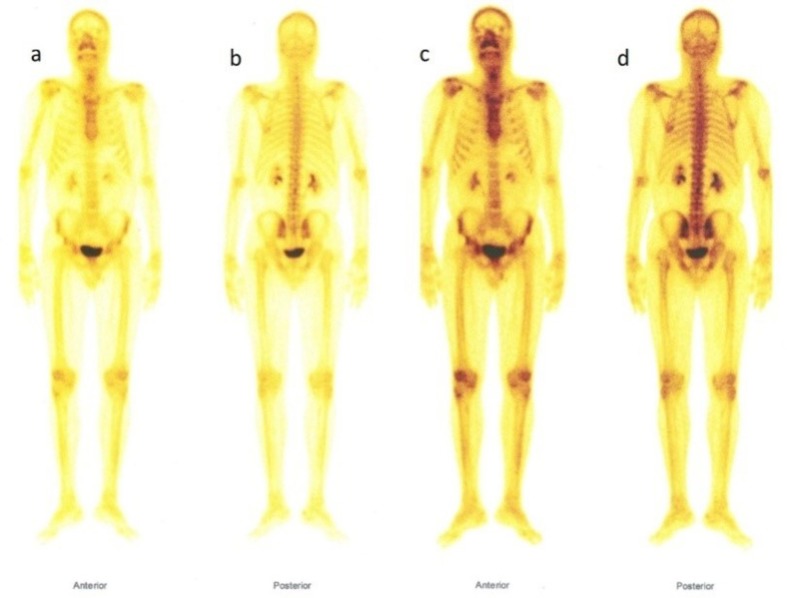
Bone scan shows no evidence of focal increased osteoblastic activity

However, the lesion showed intense FDG uptake with SUV max of 13.44 (Figure 1a, b, d) raising a suspicion of metastasis. The patient was asymptomatic. In view of this a whole body bone scan was performed on another day, 3 hours after intravenous injection of 20 mCi of Tc-99m-MDP (Methylene Diphosphonate) using a single head E-cam gamma camera (Siemens) equipped with low energy high resolution collimator. The images did not reveal any osteoblastic lesion (Figure 2).

## Discussion

Skeletal hemangiomas are asymptomatic and usually incidentally detected on CT or MRI. They are of four types of hemangioma: capillary, cavernous, arteriovenous and venous. They are commonly seen in vertebrae and ribs. Microscopically there is hamartomatous proliferation of vascular tissue. They are slow growing and cause displacement of normal bone that may appear as lytic lesion on radiograph causing corduroy cloth appearance and ‘polka dot appearance’ on CT scan due to thickened trabeculae (1). MRI shows high signal intensity due to presence of fat on T1 weighted images (T1W). T2W sequences show higher signal than T1W due to water content. After contrast injection T1W shows enhancement due to high vascularity (2).

Bone scan shows normal osteoblastic activity within these lesions (3). Rarely, these lesions may be either cold (4) or hot (5) on bone scan. FDG PET scan is usually normal in such cases (6, 7). However, in the present case FDG PET showed hot vertebra and bone scan was normal. Thus, if there is hot vertebra on FDG scan and normal bone scan of the vertebra along with characteristic signs of hemangioma on CT or MRI, biopsy is avoidable. There is a case report of ‘hot’ vertebral hemangioma (8).

^18^F-FDG PET CT has been reported to localize in malignant as well as benign skeletal lesions. Pigmented villonodular synovitis, sarcoidosis, neurofibroma, schwannoma, giant cell tumor, osteoid osteoma, histiocytosis X, chondroblastoma, enchondroma, brown tumor and non-ossifying fibroma are the benign lesions that may show FDG uptake (9, 10).

Itabashi et al reported high uptake of FDG in hemangioma of the rib and concluded that FDG may not distinguish benign and malignant rib lesions. However, they did not perform bone scan in their case (11). Similar hot hemangioma in the tibia on FDG has been reported by Cha et al. (12).

‘Hot’ cavernous hemangiomas of the ilium on FDG PET scan has also been reported with aggressive feature as post contrast inhomogeneous enhancement, thick septa and enhancing soft tissue components on CT and MR by Ko et al. (13).

Oncogenic osteomalacia could be responsible for hypermetabolism in these lesions. Fibroblast growth factor 23 is produced by benign mesenchymal cells that causes hypophosphatemia resulting in osteomalacia (14). It is postulated that tumor induced osteomalacia could be responsible for microfractures (not evident radiologically) leading to hypermetabolism in involved vertebra. However, biochemical work-up for hypophosphatemia and osteomalacia was not done in the present case.

Mesenchymal tumors associated with tumor induced osteomalacia (TIO) have been divided into: 1) phosphaturic mesenchymal tumor, mixed connective tissue type (PMTMCT); 2) osteoblastoma-like tumors; 3) ossifying fibrous-like tumors; and 4) nonossifying fibrous-like tumors (15).

^18^F-FDG PET CT, Gallium-68 Somatostatin receptor PET CT have been reported to detect mesenchymal tumors in TIO (16, 17).

Usually a hypermetabolic lesion on FDG PET scan in presence of normal bone scan and radiology invariably implies metastasis (18). 

Our case had hot 12^th^ thoracic vertebral body on FDG PET CT scan. There were two additional pointers towards hemangioma in this case. CT scan showed typical polka dot appearance and bone scan was normal suggesting a benign etiology. Clinically the patient was asymptomatic and a biopsy was avoided. Thus a discordance between FDG PET CT and Bone scan in presence of radiologic features of hemangioma is probably an indication for conservation in an asymptomatic patient.
